# Drop-In Group Medical Appointments for Patients with Asthma: A Four-Year Outcomes Study

**DOI:** 10.5402/2011/178925

**Published:** 2011-06-07

**Authors:** Myron Liebhaber, Rob Bannister, Wendy Raffetto, Zeb Dyer

**Affiliations:** Department of Allergy Immunology, Sansum Clinic, 215 Pesetas Lane, Santa Barbara, CA 93110, USA

## Abstract

Our DIGMA program was established to allow patients time to interact with an allergist, a behaviorist and an asthma educator in a group setting. Weekly meetings targeted patients with chronic asthma. DIGMAs typically last for 90 minute s and include 10 patients per session. Outcome parameters were established to assess the effectiveness of the program over a 4 year time period. Sixty four adult asthmatic patients were enrolled and followed for 4 years. Patients were seen in a group setting in groups of ten. The AQLQ test was administered each year. Spirometry, an analog self assessment scale and the ACT were administered at each visit. Forty two of the 64 patients were followed for a minimum of 3 visits to DIGMA during four years. The average baseline FVC was 85% predicted and remained unchanged. FEV1 was 78% baseline and was 77% at the last determination. Baseline rescue inhaler use was 4 per week compared to 1.5 per week at last visit. ACT scores are 18 at baseline and 19 at last visit. ER claims are 5 at one year prior to enrollment and 2 at the last year of DIGMA. Patient satisfaction improved from 30 to 34 at the last visit. This was an effective, multidisciplinary asthma intervention that focused on behavior. It fulfilled the goals of asthma care as described by the 2007 NAEPP guidelines.

## 1. Introduction

Drop-In Group Medical Appointments (DIGMAs) were provided for adult patients who are encouraged to attend group discussions. This was developed in the early 1990s to supplement routine visits for geriatric care. DIGMAs have been developed for diabetes, hypertension, well-child care, fibromyalgia, and functional bowel disease [1]. Prior studies using the group model have reported improvements in hemoglobin A1C, diabetic knowledge, and quality of life [2–4]. This model has been shown to increase physician and staff productivity as well as decrease healthcare cost. Our weekly DIGMAs target those patients with chronic asthma. DIGMAs typically last for 90 minutes and involve up to 10 patients [5]. Family members are also encouraged to attend. The physician addresses medical concerns, the behaviorist facilitates group discussion and psychosocial issues, and the asthma educator addresses specific questions regarding monitoring and equipment. This allows for patient education and self-management instruction without the limitation of a formal didactic presentation.

Our program, which we renamed Doctor Interactive Group Medical Appointments, DIGMAs was designed to allow the physician to evaluate asthmatic patients on a weekly basis and provide disease education. In addition, these group visits allow the patients to interact with one another in a mutually supportive environment. Outcome parameters were established to assess the effectiveness of the program for 4 years.

## 2. Methods

Sixty-four adult asthmatic patients were enrolled in our DIGMA program and followed for four years. These enrollees were identified as high utilizers of the health care system. Groups of ten patients per week were seen together. Patients were seen in 3 steps: vital signs and spirometry recorded by a nurse, an interim brief history, and physical exam performed by the physician, and a group session usually lasting 60–90 minutes, supervised by the behaviorist. 

The Asthma Quality of Life Questionnaire (AQLQ) was utilized to benchmark the patient's perception of their disease prior to their first DIGMA visit and at one year after the initial group appointment [6]. AQLQ is a validated instrument that measures responses in 4 domains: activity limitation, symptoms, emotional function, and environmental exposure. The patients performed spirometry testing and completed an analog scale used to measure compliance and satisfaction with care (Figure 3). An Asthma Control Test (ACT) was also administered at each visit [7]. ACT results were then reviewed with the group, comments were elicited, and concerns addressed. These outcomes were reported after four years of surveillance.

## 3. Results

**Figure 1:**
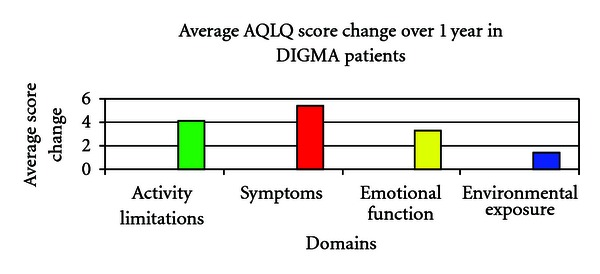
Asthma Quality of life Questionaire.

**Figure 3:**
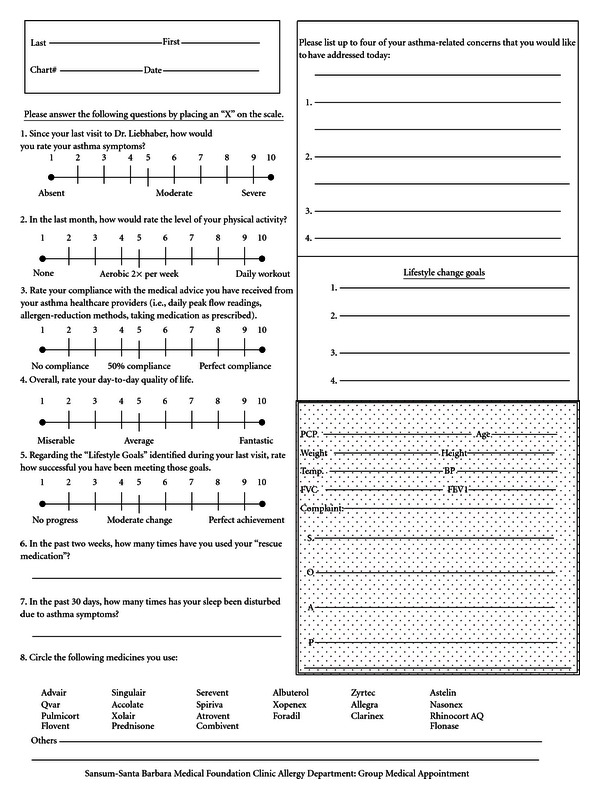


Forty-two patients continued the DIGMA program for four years and were considered regular attendees (Table 1). The remaining twenty two patients were considered intermittent attendees. Twenty-six patients completed the baseline and 1 year postbaseline AQLQ. The AQLQ scores were improved by 373 points at the end of the first year. The changes in scores for each domain can be seen in Table 2. The average improvement per patient in each domain of AQLQ scores can be seen in Figure 1. Although there was no control group, baseline data on each patient was analyzed one year prior to study entry. 

The four-year outcomes data showed the following: average baseline FVC was 85% predicted with the last available FVC unchanged at 85% predicted. The average baseline FEV1 was 78% predicted with the last available FEV1 77% predicted. Baseline ACT scores were 18 at the time the test became available and were slightly improved at 19 at the last DIGMA appointment. The analog scale provides a basis for psychosocial inquiries, rescue inhaler use, and nocturnal awakening. The baseline patient satisfaction score was 30.46 and improved to 34.43 at the last determination. This was an overall 12% improvement in patient perception (Table 1). Average baseline rescue inhaler use was 4 per week compared to 1.5 per week at the last determination. Nocturnal awakening average was 5.5 per month compared to 1 per month at the last determination. Average baseline ER and hospital claims data during the year prior to enrollment was 5 compared to 2 claims at the end of the last year of the four-year period for the forty-two patients who continued DIGMA (Table 1). 

## 4. Discussion

DIGMA is a targeted intervention that fulfills the treatment goals of asthma care, as described by the National Asthma Education and Prevention Program (NAEPP) Guidelines [8]. These treatment goals include symptom and exacerbation prevention, the maintenance of pulmonary function, optimized activity levels, meeting expectations of satisfaction with asthma care, and the provision of optimal pharmacotherapy. Each goal had a corresponding measurable outcome. Asthma symptoms were monitored by the ACT, pulmonary functions were monitored by serial spirometry, activity level was monitored by the AQLQ test, exacerbations were monitored by claims data, and satisfaction with care was monitored by the analog scale. All survey scores were either unchanged or slightly improved after a four-year time period. Our DIGMA results agreed with a previous report of chronically ill older adults in a Health maintenance organization (HMO) involving 9 facilities and 19 physicians. These investigators found no difference in the number of outpatient visits, but significantly fewer emergency visits and hospital admissions [9]. 

During the first visit confidentiality agreements and consents were signed according to the Health Insurance Portability and Accountability Act (HIPAA). The treating physician provides a one-to-one visit prior to the group intervention. Visits were billed and reimbursed as individual office visits using existing current procedural terminology (CPT) codes ranging from 99212 to 99215. Spirometry codes are also billed as 94375 [10].

We met the goals of asthma therapy by using appropriate outcomes measurements. Motivating factors and barriers to healthcare access can affect outcomes [11]. DIGMA removed these barriers and provides extra value by allowing patients to attend any session at any time, providing easy access to spirometry, vaccines, prescription renewals, and health care advice as often as weekly if necessary. This program focused on changes in behavior. The patients completed the analog evaluation at each visit which led to a discussion of satisfaction and compliance with care. Psychosocial factors were discussed for each patient by the behaviorist. Often, individual patient concerns were discussed as a group leading to a dialog of experiences by other participants. One of the primary goals was to enhance education, awareness, and disease understanding without the limitation of a formal lecture. Outcomes data confirmed that this was an effective multidisciplinary asthma intervention. 

## Figures and Tables

**Figure 2:**
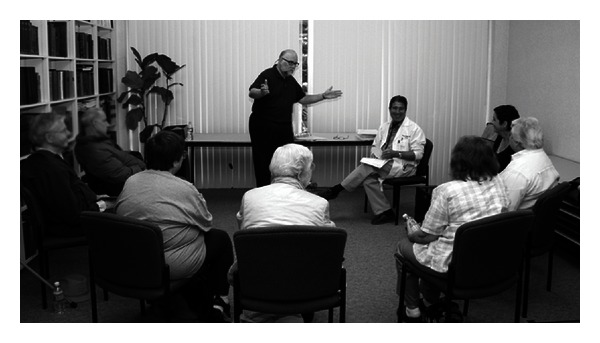


**Table 1:** 42 adult patients 6/2004–6/2008 Sansum Clinic Asthma DIGMA^3^.

Outcome	Baseline	Last visit	% Change
Patient satisfaction ^1^	30.46	34.43	+12
ACT^2^	18	19	+6
FVC % predicted	85	85	0
FEV1%predicted	78	77	−2
Ave Rescue Medication	4	1.5	−50%
Nocturnal wakening/month	5.5	1	−72%
ER/Hospital utilization**^4^**	5	2	−60%

^1^Analog patient satisfaction score (Table 1).

^2^Asthma Control Test.

^3^Doctor-Integrated Group Medical Appointments.

^4^Based on claims data from 17 HMO patients.

**Table 2:** AQLQ scores per domain baseline and 1-year after.

Domain	Baseline results	1-year after	Difference	Missing responses
Activity Limitations	1153	1261	108	48
Symptoms	1486	1628	142	13
Emotional function	606	693	87	5
Environmental exposure	550	586	36	5
